# A Military Hospital a Hundred Years Ago

**Published:** 1898-09-03

**Authors:** 


### A MILITARY HOSPITAL A HUNDRED YEARS AGO.

### INSTRUCTIONS TO REGIMENTAL SURGEONS FOR REGULATING THE CONCERNS OF THE SICK AND OF THE HOSPITAL.

Such is the quaint title of a somewhat interesting and venerable little volume dated 1808. It consists of only seventy-nine pages, and as these are English and its German translation alternately, the regimental doctors of those days were not overburdened with rules and regulations.

The lowest rank of surgeon was what is curiously termed "Hospital Mate." Nothing is said of the duties of this official. There were several intermediate grades of medical officers up to the rank of Inspector-General of Army Hospitals.

It appears that each regiment, as a rule, had three medical officers, the regimental surgeon and two assistant surgeons, who had the regimental hospital under their immediate control, subject to the inspector-general and his deputies. The regimental surgeon was supposed to keep all books, whether medical notes referring to the cases, or accounts of the management of the hospital, and the commanding officer of the regiment inspected them all from time to time. The surgeons were directed to visit the hospital "at least twice a day," and to keep a register of all cases admitted or discharged, with their treatment, diet, and disease.

When the regiment was stationed in permanent barracks a properly equipped hospital was provided, the surgeon being responsible for all stores and furniture. He is particularly told "not to be inattentive to the state of his own regimental hospital equipment, and to frequently expose the bedding to the open air, to prevent injury by damp, or other causes." At such times when the regiment was billeted out in quarters he had to choose and rent a suitable house for a hospital.

The barrack department furnished the straw to fill the beds in the permanent hospital, but in a hired one it had to be purchased by the surgeon, who charged for it in the weekly returns. Bedsteads formed part of the equipment of the barrack hospitals, but in hired ones the bed was placed on one or two straw mats instead, and in tent hospitals the men were put on temporary bedsteads made from faggot wood drawn from the commissary, which, with a few nails, was considered all that was necessary.

The number of "hospital servants" for a battalion of 500 men (battalions were smaller in those days) was one nurse (female) at 1s. a day, one sergeant at 9d., and one orderly at 4d. a day; this 9d. and 4d. being for extra duty, in addition to their regular pay. Truly a magnificent staff, but one which represented the maximum expense to which the public were to be put except in cases of great need and "undue sickness," when extra help might be obtained and accounted for in the weekly returns. If the battalion was split up into three detachments, the services of the costly nurse were to be dispensed with, and three orderlies told off to take her place. The duties of the hospital sergeant were to take charge of all bedding, utensils, and stores, and be responsible to the surgeon for them. He had to go round the wards frequently, give medicine and food, and see that the nurse and orderly did their work properly, and that the wards were kept clean and tidy; in fact, perform what is now known as sisters' duties. The nurse and orderly shared the duty of nursing between them, and, when "not too much engaged with the sick," the latter had to wash the bedding. Each patient was provided with a clean shirt twice a week, and clean sheets once a fortnight, or oftener if required, and each man had a bed to himself. On admission, every patient was to have "his whole body made extremely clean with warm water and soap," and all the men were to have their hands and faces washed and their hair combed and *tied* before the doctor's visit in the morning. The wards were ordered to be "frequently fumigated" with nitric acid, and detailed directions are given of the method.

The surgeon was to be in possession of a complete set of instruments, provided at his own expense. He had to send in weekly returns of the expenses of the hospital, monthly ones about the sick, and half-yearly ones on the necessary stores and medicines. He was specially told that "the inoculation of the cow-pox was to be constantly practised." Many directions in this book are rather vague. For instance, would the term "constantly" be taken to mean every day, once a month, or how often? Again, the wards had to be "frequently" fumigated; that is also capable of very elastic interpretation, a fact that the very limited hospital staff would be only too glad to avail themselves of, one would imagine. In fact, it seems highly probable that many of the rules would be more honoured in the breach than the observance. Below is the regulation table of diets:—

| Meals. | Full Diet. | Half Diet. | Low Diet. | Spoon or Fever Diet. |
|---|---|---|---|---|
| Breakfast ... | 1 pt. of milk porridge or rice gruel. | 1 pt. of milk porridge or rice gruel. | 1 pt. of milk porridge or rice gruel. | Tea. |
| Dinner ... ... | ¾ lb. of meat, 1 lb. of bread. | ½ lb. meat, ½ lb. of potatoes, ½ lb. of bread. | ¼ lb. meat, or same made into weak broth; ½ lb. potatoes, ½ lb. bread. | 1 lb. bread made into panado, or pudding with as much milk, and sago. |
| Supper ... ... | 1 pt. of broth made from the meat. | 1 pt. of broth made from the meat. | 1 pt. of milk porridge or rice gruel. | Tea. |

Remarks.—All extra diet to be given at the discretion of the surgeon, but it must be stated or charged in the proper able of the weekly returns. Wine used in panado, sago, or any other kind of food, must be particularly specified in the wine return.

N.B.—The milk porridge is supposed to consist of 3 parts gruel and 1 part milk. The spoon diet is adapted to fevers and such cases as will not allow of any excitement from animal food in the shape of broth or otherwise. When beer is judged necessary the expense is not to exceed 1d. per man.

